# 1153. ESBL Producing *E. coli* Urinary Tract Infections in Children: Is Carbapenem Always Necessary?

**DOI:** 10.1093/ofid/ofab466.1346

**Published:** 2021-12-04

**Authors:** Ruiz Cabrales Diva del Carmen, Gabriel Ivan Narvaez Oviedo, Juan Pablo Londono-Ruiz, Ivan Felipe Gutiérrez Tobar

**Affiliations:** 1 Universidad El Bosque, Bogotá, Distrito Capital de Bogota, Colombia; 2 Clinica Infantil Colsubsidio, Clínica Infantil Santa María del Lago, Bogotá, Distrito Capital de Bogota, Colombia

## Abstract

**Background:**

Urinary tract infections (UTI) are common in children with a prevalence of 5% in infants. UTI are the main reason for beginning antibiotics in children’s hospitals and *E. coli* is approximate 80% of urinary pathogens. Extended-spectrum beta-lactamases (ESBL) producing *E. coli* are a common concern in daily practice. Carbapenems, especially ertapenem are the choice for the treatment in some hospitals, but aminoglycosides or trimethoprim and sulfamethoxazole are options for carbapenem saver. The aim of this study was comparing the clinical outputs in ESBL producing *E. coli* ITU in children treated with ertapenem or amikacin.

**Methods:**

We designed a quasi-experimental study. In 2018 the antimicrobial stewardship program begins the use of amikacin for non-septic UTI for ESBL producing E. coli. Before this recommendation the use of ertapenem was common. We use WHONET 5.6 to identify ESBL producing *E. coli* UTI between 2016 and 2020. We analyzed the information using R 4.0.3.

**Results:**

We analyzed 162 clinical records. 89 in ertapenem group, 45 in amikacin group, 23 in other treatments (TMP-SMX, meropenem) and 5 patients that received empirical treatment (Cefazolin) with clinical improvement and ambulatory management. The initial clinical and paraclinical variables was similar between two groups, only meropenem was more frequent in amikacin group as empiric treatment (table 1). Amikacin group received for media 7.4 days of antibiotic therapy (IQR 7-7.5) and ertapenem 8.2 days (IQR 7-10) (p value 0.049). The mortality, PICU requirement, mechanical ventilation and inotropic requirement was similar an both groups (Table 2). In amikacin group the median length of stay was 7.2 days (IQR 4-9) and in ertapenem group was 9 days (IQR 6-10). No significant adverse effects were documented in any group.

Table 1. Patient’s characteristics in both groups.

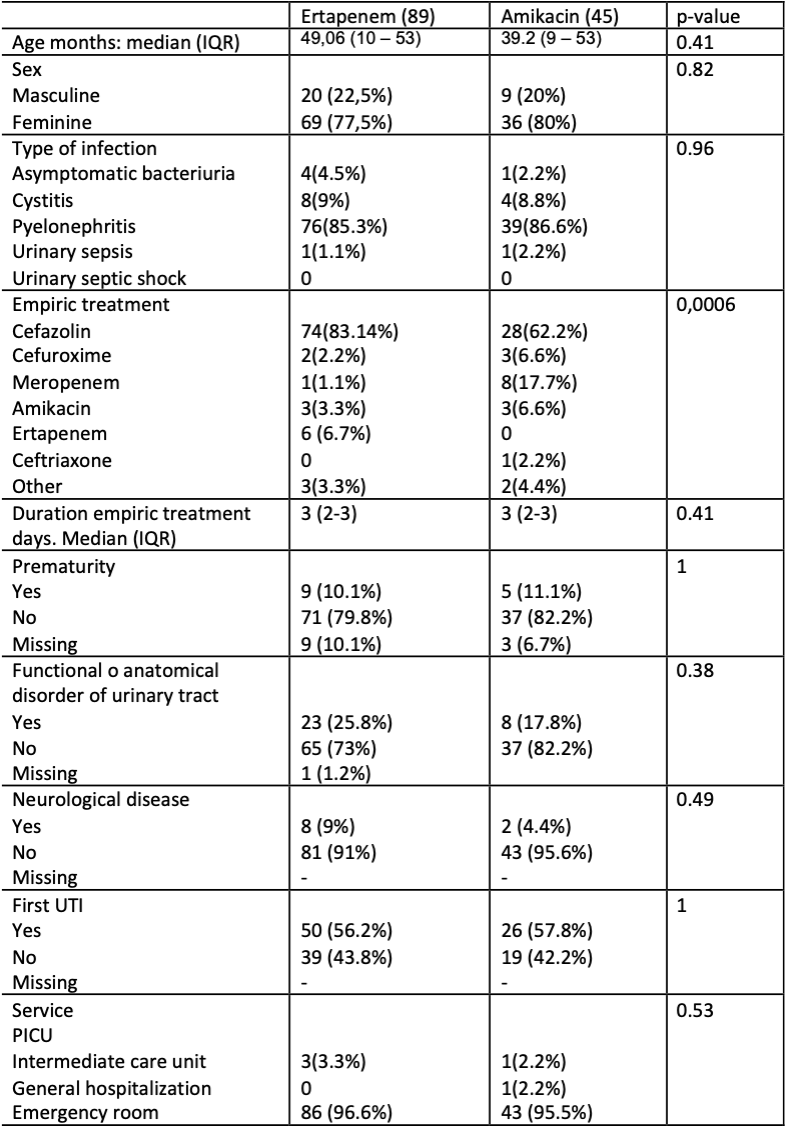

Table 2. Patient’s Clinical outcomes in both groups

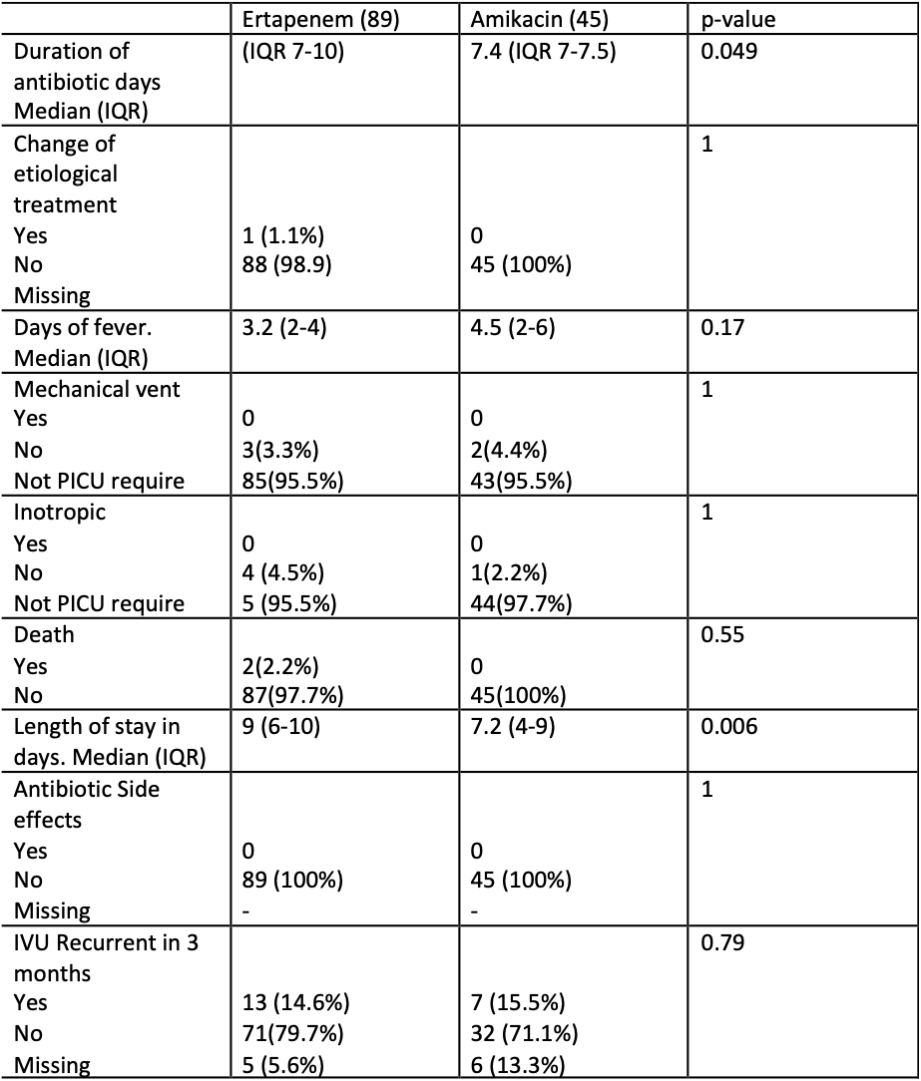

**Conclusion:**

The use of amikacin in ESBL producing *E. coli* UTI in children have similar clinical outputs that ertapenem. The use of amikacin could decrease de hospitalization time.

**Disclosures:**

**Ivan Felipe Gutiérrez Tobar, n/a**, **Pfizer and MSD** (Advisor or Review Panel member, Research Grant or Support, Speaker’s Bureau, Has received support from Pfizer and MSD for participation in congresses and has received conference payments from Pfizer)**Pfizer and MSD** (Speaker’s Bureau, Other Financial or Material Support, Has received support from Pfizer for participation in congresses)

